# No Viral Association Found in a Set of Differentiated Vulvar Intraepithelial Neoplasia Cases by Human Papillomavirus and Pan-Viral Microarray Testing

**DOI:** 10.1371/journal.pone.0125292

**Published:** 2015-04-20

**Authors:** Ozlen Saglam, Erik Samayoa, Sneha Somasekar, Samia Naccache, Akiko Iwasaki, Charles Y Chiu

**Affiliations:** 1 Department of Pathology, Moffitt Cancer Center, Tampa, Florida, United States of America; 2 Department of Laboratory Medicine, University of California San Francisco, San Francisco, California, United States of America; 3 UCSF-Abbott Viral Diagnostics and Discovery Center, San Francisco, California, United States of America; 4 Howard Hughes Medical Institute, Department of Immunobiology, Yale University, New Haven, Connecticut, United States of America; 5 Department of Medicine, Division of Infectious Diseases, University of California San Francisco, San Francisco, California, United States of America; International Centre for Genetic Engineering and Biotechnology, ITALY

## Abstract

Vulvar Intraepithelial Neoplasia (VIN) is the precursor lesion of Vulvar Squamous Cell Carcinoma (VSCC), and the differentiated type (dVIN) is more frequently observed in relation to VSCC. In contrast to usual-type VIN (uVIN), which is related to infection by human papillomavirus (HPV), a germline mutation in the *p53* gene is thought to be associated with ~90% of dVIN cases. To date, no infectious agent has been identified in association with dVIN, and studies investigating this possibility have been hindered by the difficulty in accurately diagnosing dVIN from small biopsies. Here, we used immunostaining for p16^ink4a^, a biomarker for HPV infection, to study 14 uVIN high-grade VIN and 14 dVIN cases, and to select 10 dVIN cases to broadly screen for all known viruses using a pan-viral microarray platform (ViroChip). All of the uVIN tissue samples, including 8 warty and 6 basaloid cases, showed positivity with the p16^ink4a^ immunostain. The staining pattern was full-thickness for all except two cases in which positive staining was localized in the lower 1/3 of the epidermis. In contrast, immunostaining for p16^ink4a^ was negative in all dVIN cases. ViroChip analysis of 10 pure dVIN samples confirmed the absence of human papillomavirus subtypes or any other virus with the exception of a single sample that showed a weak microarray signature to a porcine herpesvirus. Follow-up PCR testing of the sample was negative for herpesvirus, and in-depth metagenomic next-generation sequencing revealed only sequences corresponding to non-pathogenic viral flora and bacterial contamination. In this study, we demonstrated lack of a virus association in 10 dVIN cases. Alternative pathways for carcinogenesis such as the *p53* mutation should be considered for investigation of potential treatment options in dVIN.

## Introduction

The incidence of Vulvar Intraepithelial Neoplasia (VIN), a precursor lesion of Vulvar Invasive Squamous Cell Carcinoma (VSCC), is increasing according to Surveillance Epidemiology and End Result Data [1]. VIN is divided into two major groups based on morphologic and known etiopathogenic pathways. Usual-type VIN (uVIN) comprises more 90% of all VIN cases and has two subtypes, warty and basaloid VIN [2]. The uVIN tissue samples are graded on a three-tiered scale from mild to severe depending on the proportion of epithelium containing abnormal proliferation. Although International Society for the Study of Vulvovaginal Disease proposed to change the three-tiered grading system in 2005 [3], the traditional scale is still widely used in clinical practice. All uVINs are strongly associated with human papillomavirus (HPV) infections. HPV 16 is the most common viral agent identified in both VIN and VSCC. Other HPV types, such as 18, 31, 33, and 45, have also been reported in varying percentages [4]. However, despite nearly 4/5 of VIN cases testing positive for HPV [5], the association of VSCC cases with HPV infection is not strong, with only 1/5 to 1/2 of VSCC cases linked to HPV infection.

Differentiated Vulvar Intraepithelial Neoplasia (dVIN) is a high-grade VIN and is the second, less common type, constituting only 2–10% of VIN cases [6]. Establishing the diagnosis of dVIN is challenging especially from small biopsies. Characteristic morphologic features include thickened epidermis with elongated rete ridges and prominent intercellular bridges. Keratin pearl formation is commonly observed. Keratinocytes are enlarged with large vesicular nuclei and abundant, eosinophilic cytoplasm [7]. In contrast to uVIN, dVIN is not a frequent diagnosis as an isolated lesion, and is usually observed around invasive squamous cell carcinoma and lichen sclerosus in vulvectomy specimens. Immunohistochemical staining for the tumor suppressor *p53* gene has been reported to label dVIN in ~90% of cases, with p53-positive cells typically extending above the basal layer into higher levels of the epidermis [8]. However, a number of dVINs do not show mutations of *p53* or are associated with deletions of *p53*, suggesting that other causes, potentially infectious, may play a role in at least a subset of dVIN cases. Given that dVIN is a more common precursor of invasive VSCC than uVIN, there is a need for better diagnostic markers for these diseases that are both highly sensitive and specific.

The ViroChip is a pan-viral microarray platform consisting of ~60,000 probes and has the capacity to simultaneously detect all ~2,500 known viruses in GenBank as of 2011 in a single assay [9,10]. Novel viruses and strain variants can also be identified on the basis of homology to conserved genes [11,12]. In respiratory secretions, the sensitivity of detection for the platform has been shown to be comparable to that of specific viral PCR [13,14]. In addition, viruses such as titi monkey adenovirus and the XMRV gammaretrovirus have been successfully detected using the ViroChip in lung and prostate tissues, respectively [11,15], despite the potentially confounding presence of high host background DNA in tissue samples. Unbiased next-generation sequencing is another genomics-based strategy for broad-spectrum microbial detection that has proven to be useful for viral discovery [16,17] and infectious disease diagnosis in the clinical setting [18]. Here we present the use of both approaches, coupled with p16^ink4a^ immunostaining [19] and PCR [20] for HPV identification, to investigate a potential role of viral infection in dVIN.

## Methods

### Sample Collection

**Fig 1:**
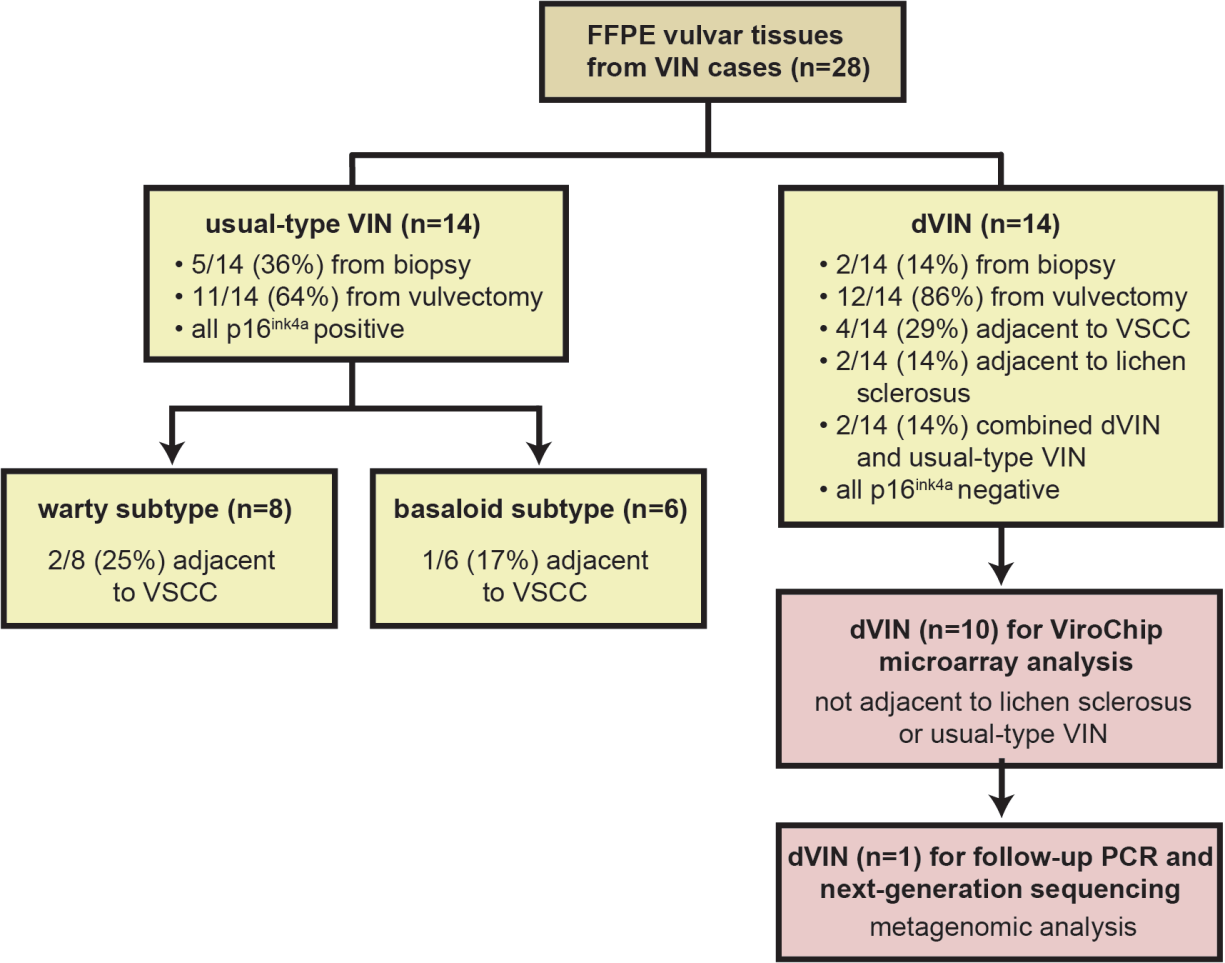
Schematic flowchart showing testing of FFPE vulvar tissues from 28 cases of high-grade differentiated VIN (dVIN) and usual-type VIN (uVIN). Histological (yellow boxes), p16^ink4a^ immunostaining (yellow boxes), and genomic (pink boxes) analyses were performed. Abbreviations: FFPE, formalin-fixed, paraffin-embedded; VIN, vulvar intraepithelial neoplasia; VSCC, vulvar squamous cell carcinoma; PCR, polymerase chain reaction.

After approval was obtained from the Yale University institutional review board (IRB), high-grade VIN (classified as VIN3, severe dysplasia) cases were retrieved from the Yale Pathology Department archives. The cohort consisted of 14 dVIN and 14 uVIN cases (Fig 1). No consent was obtained because samples were collected and data analyzed in an anonymous fashion. Samples were analyzed by microarray, PCR, and NGS under protocols approved by the University of California, San Francisco IRB (IRB #11–05519).

### p16^ink4a^ Immunohistochemistry

Five μm sections were cut from paraffin blocks and mounted on charged slides. They were deparaffinized in xylene and rehydrated through graded concentration of ethanol. Following that, they were immersed in citrate buffer pH 6.0 at 95–101 degrees C for 20 minutes. Once the slides were cooled down they were rinsed & placed in Tris buffered saline. Endogenous peroxidase is quenched with 3% Hydrogen peroxide. The primary antibody p16^ink4a^ (BD Biosciences, catalog # 551154. San Jose, CA) was applied at a dilution of 1:200. The antibody was detected with Envision+ from DAKO (catalog# K4001. Carpentaria, CA) then visualized with Diaminobenzidine and counterstained with hematoxylin. Slides were then dehydrated, cleared & mounted with resinous mounting media.

### ViroChip Analysis

For ViroChip analysis, nucleic acid was extracted from 10um Formalin fixed Paraffin Embedded (FFPE) sections. Extractions were performed by scraping off the tissue from 2 to 4 slides (depending on the size of tissue) and incubating the tissue in Buffer G2 and proteinase K (Qiagen, Hilden Germany) overnight. After tissue digestion, debris was pelleted by centrifugation, and 200 μL of the supernatant was used for total nucleic acid extraction using the EZ1 DNA universal tissue kit (Qiagen, Alameda, CA).

Samples were randomly amplified to generate a cDNA library as previously described, and examined by 2% agarose gel electrophoresis to assess the quality and size distribution of the libraries. The samples were then labeled with Cy3 fluorescent dye and measured on a spectrophotometer to ensure adequate dye incorporation and cDNA concentration. All samples passed this quality check (QC) and were hybridized overnight to a custom 70mer ViroChip microarray (Agilent, Santa Clara, CA). The current version of the ViroChip (v5.0) consists of 59,436 probes derived from all viral sequences in GenBank as of December 2011 (NCBI GEO microarray accession number GPL11662). Following washing and scanning according to the manufacturer’s protocol, microarray data were analyzed using the in-house developed software package Viroview, which enables manual visualization of probe intensities, formatting of arrays for cluster analysis [15,21], and generation of ranked Z-score tables. Raw microarray intensities were background-corrected by removal of probes with raw intensity >100 from an age-/sex- matched negative control sample (normal vulvar skin biopsy; Table 1, “Normal”). For Z-score analysis, a previously screened set of 10 negative human respiratory (nasal swab) samples was used to generate normalized median intensity values for the remaining probes. Viral hits were considered positive by Z-score analysis if there were at least 5 positive probes corresponding to a given viral species out of the ranked top 50 that mapped to ≥3 regions of the viral genome [11]. Heat maps of sum-normalized probe intensities were constructed using Clustal v3.0 [21] and visualized using Java Treeview [22].

**Table 1:** Ranked Z-score analysis of ViroChip microarrays corresponding to dVIN samples and controls for virus identification.

Sample Name	Positive ViroChip Signature*	Number of Hits out of Top 50	# of Distinct Locations on the Genome**
dVIN-Sample1	Suid herpesvirus 1	5	3
dVIN-Sample2	None	N/A	N/A
dVIN-Sample3	None	N/A	No
dVIN-Sample4	None	N/A	No
dVIN-Sample5	None	N/A	No
dVIN-Sample6	None	N/A	No
dVIN-Sample7	None	N/A	No
dVIN-Sample8	None	N/A	No
dVIN-Sample9	None	N/A	No
dVIN-Sample10	None	N/A	No
Normal	None	N/A	No
Positive Control	Human papillomavirus 18	16	6

*Criteria: ≥5 hits out of top 50 probes (10%) and probe hits mapped to ≥3 distinct locations on the viral genome.

**Distinct locations: mapped probe locations on the viral genome separated by at least 5% of the total genome length.

### PCR

PCR confirmation of HPV signatures was performed using a previously published PCR assays targeting the conserved L1 gene of human papillomaviruses (GP5+/GP6+ PCR) [20]. We also designed primer sets targeting each of the 4 papillomavirus subtypes represented in the low-intensity clusters that were observed by ViroChip microarray analysis of dVIN samples (Table 2). For each primer set, one of the primers was designed directly from the microarray probe sequence, and the other primer was designed from the genomic sequence corresponding to the specific papillomavirus subtype.

**Table 2:** Number of next-generation sequencing (NGS) reads at each step of the SURPI bioinformatics pipeline for pathogen identification.

Primer Name	Sequence	HPV-Specific Target
VIN5-HPV34-6433F	GGT TGC AAA AGG CCC AGG GAC	HPV34
VIN5-HPV34-6620R	CAG GTC ATA CTC TTC TGC ATG TCT G	HPV34
VIN5-HPV81-7287F	CAT CTA AAC TGG TGG AAG GAG GTG	HPV81
VIN5-HPV81-7081R	GAC ACA GCA AAA GAC CGG GTG C	HPV81
VIN4-HPV83-1636F	GAA CCA CCA AAA ATA CGT AGC GGG	HPV83
VIN4-HPV83-1837R	GGT CGC TTT CCT CCT GGA TAT C	HPV83
VIN9-HPV83-6470F	GGT GAT TGT ATG TTC TTT TGC CTC CG	HPV83
VIN9-HPV83-6697R	TGG GCA CGA TGC AGC CAG TAG	HPV83
VIN9-HPV7-4560F	ATG GGG CAG CAT GGG AGT GTT	HPV7
VIN9-HPV7-4739R	TCA GTG GGG GCG ACA GTA TCC	HPV7

To investigate presumed cross-contamination of dVIN sample 1 from an unrelated HHV3-positive cerebrospinal fluid (CSF) sample processed in the same NGS run, primers VIN1-HHV3-F (5’-TCGTACCGATGGAGGGTTAC-3’) and VIN1-HHV3-R (5’-GACTTACGCGTTTCGGTTTC-3’) were designed from the single HHV3 read found in the dVIN sample 1 NGS data. PCR was performed using the TaKaRa Ex Taq DNA Polymerase Kit (Takara Bio, Kyoto, Japan) and the following cycling conditions: 95°C for 5 min, then 40 cycles of 94°C for 30s, 55°C for 30s, 72°C for 30 s, and a final extension at 72°C for 5 min. To test dVIN sample 1 for Suid herpesvirus 1, primers VIN1-SuHV1-F (5’-CGTGATCTGCGTGCTCTG-3’) and VIN1-SuHV1-R (5’-AGCTCCCGAACAAGATCGTC-3’) were designed from two SuHV1 probes showing high-intensity on the dVIN sample 1 ViroChip microarray. PCR conditions using the Qiagen OneStep RT-PCR Kit were as follows: 50°C for 30 min, followed by 95°C for 15 min, then 40 cycles of 94°C for 30s, 55°C for 30s, 72°C for 30 s, and a final extension at 72°C for 5 min.

### Metagenomic Next-Generation Sequencing

Next-generation sequencing for pathogen detection was performed as previously described [23]. Briefly, NGS libraries were constructed from total nucleic acid using a modified TruSeq protocol. Library size and concentration were determined using the BioAnalyzer High-Sensitivity DNA kit (Agilent, Santa Clara, CA) and Kapa Universal qPCR kit (Kapa Biosystems, Woburn, MA), respectively. Samples were sequenced on an Illumina MiSeq instrument using 200/135 base pair (bp) paired-end sequencing. NGS data was analyzed using the SURPI bioinformatics pipeline for pathogen detection [24]. The SURPI pipeline first identifies and computationally subtracts human host sequences using the nucleotide aligner SNAP [25], followed by SNAP identification of viruses, bacteria, fungi, and parasites by comprehensive mapping of all remaining reads to the National Center for Biotechnology Information (NCBI) nucleotide (nt) reference database (NCBI nt). For discovery of novel viruses, remaining unmatched reads and *de novo* assembled contigs are then mapped to a viral protein database using the translated nucleotide aligner RAPSearch [26]. Viral hits by SURPI were further curated for accuracy by manual inspection and BLAST alignment to the NCBI nucleotide (nt) and amino acid (nr) reference databases.

## Results

**Fig 2:**
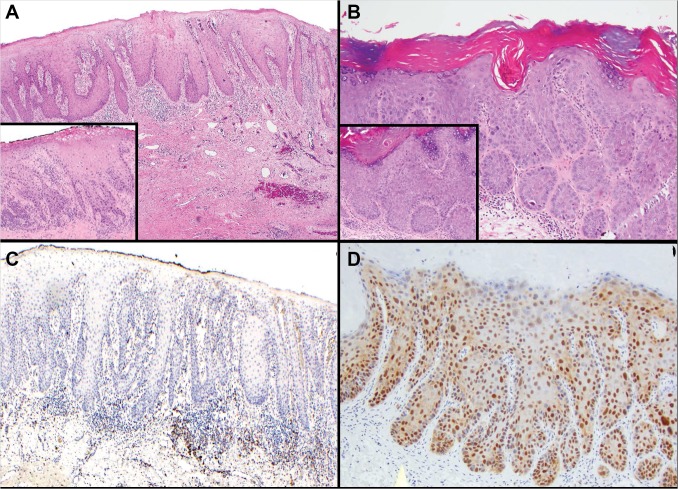
Histology and p16^ink4a^ immunostaining patterns of high-grade dVIN and uVIN samples. (A) dVIN, showing characteristic elongation and anastomosis of rete ridges. The epithelium shows enlarged keratinoocytes with abundant eosinophilic cytoplasm. The inset displays prominent cytologic atypia localized to the lower 1/3 of the epithelium. (B) uVIN, warty subtype. A spiked surface epithelium with hyperkeratosis and hypergranulosis is visualized. The inset displays full-thickness cytologic atypia. (C) p16^ink4a^ immunostaining is negative in dVIN. (D) Full-thickness p16^ink4a^ immunopositivity is seen in high-grade uVIN. Abbreviations: dVIN, differentiated vulvar intraepithelial neoplasia; uVIN, usual-type vulvar intraepithelial neoplasia.

The median age for patients with dVIN and uVIN was 77 and 53 years, respectively. All 14 dVIN cases (Fig 2A) showed elongation and anastomosis of rete ridges, dyskeratotic cells within the epidermis, at least focal parakeratosis, and significant cytologic atypia in the lower 1/3 of the epidermis (Fig 2A, inset). None had keratin pearl formation. All of the warty (Fig 2B) and basaloid uVIN cases showed positivity for immunostain p16^ink4a^ (Fig 2D). There was full-thickness epithelial staining for all except for two basaloid-type VIN cases, in which p16^ink4a^ positivity was localized to the lower 1/3 of the epidermis. In contrast, no p16^ink4a^ expression was observed in 12 dVIN cases (Fig 2C). The two combined dVIN and uVIN cases (Fig 1) showed positivity for p16^ink4a^ immunostaining in uVIN dysplastic tissues and minimal blushing in the dVIN component.

**Fig 3:**
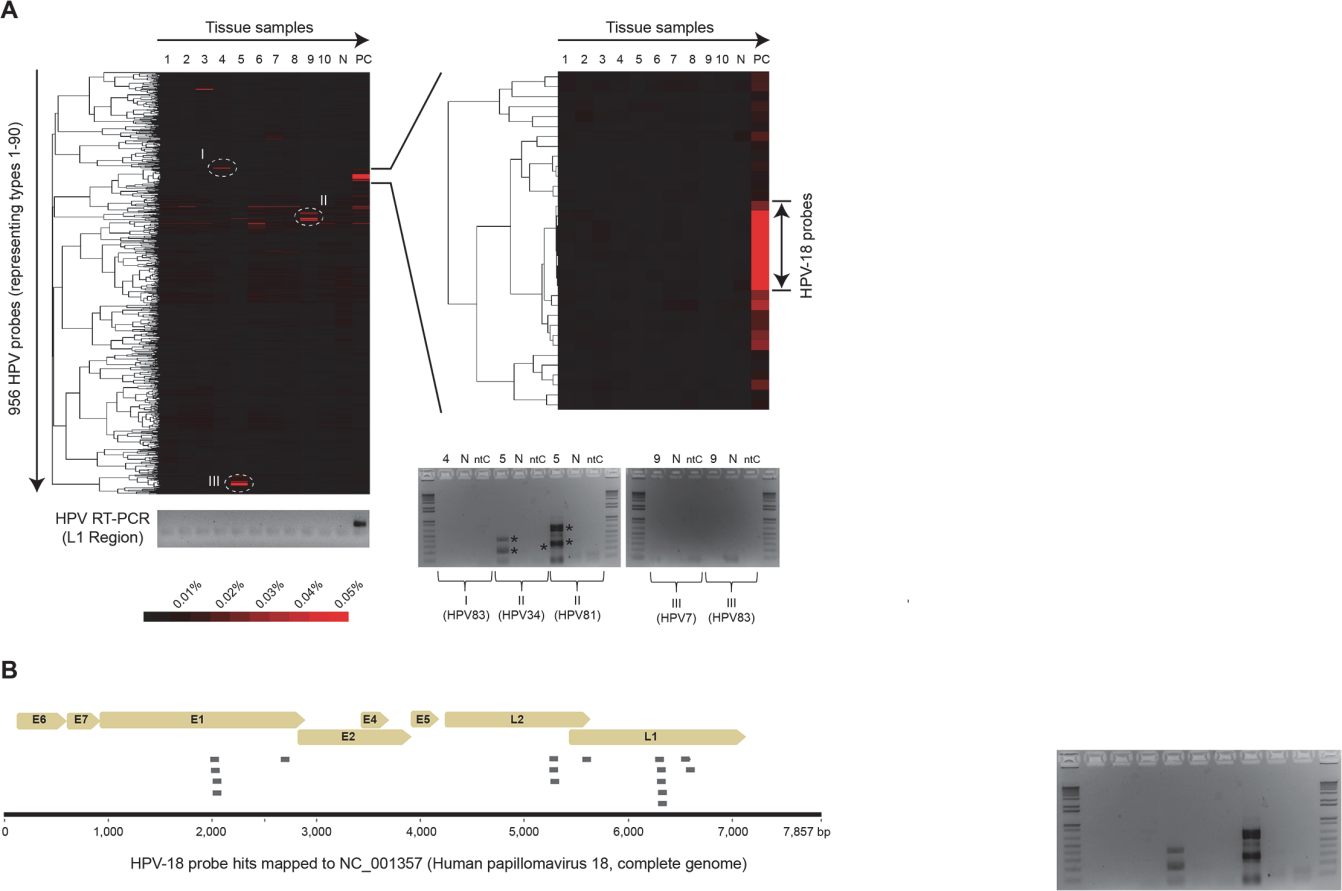
Pan-viral analysis of high-grade dVIN samples. (A) Tissue samples (x-axis) were analyzed using the ViroChip, a pan-viral DNA detection microarray. The cluster heat map of HPV probes (y-axis) shows only one strong papillomavirus cluster corresponding to the HPV18 cervical cancer positive control (PC). Other scattered clusters corresponding to the dVIN samples (tissue samples 1–10) consisted of lower-intensity probes representing multiple papillomavirus subtypes, including HPV7, HPV34, HPV81, and HPV 83 (clusters I, II, and III). Confirmatory PCR testing from the HPV L1 region using conserved primers was negative for all of the dVIN samples (gel, left). PCR testing using primers specific for each HPV subtype was also negative (2 gels, right), with the exception of bands in dVIN sample 5 that were cloned and sequenced as *Pseudomonas aeruginosa* (asterisks), and attributed to bacterial surface contamination of the FFPE block. Thus, the dVIN samples were deemed negative for HPV infection. The red color bar denotes the normalized magnitude of hybridization intensity. (B) Mapping of 16 HPV18 probes out of the top 50 (by ranked Z-score analysis) to the HPV18 genome in the cervical cancer positive control. Abbreviations: HPV, human papillomavirus; VIN, vulvar intraepithelial neoplasia; N, normal skin vulvar biopsy; PC, positive control; ntC, no template control.

**Fig 4:**
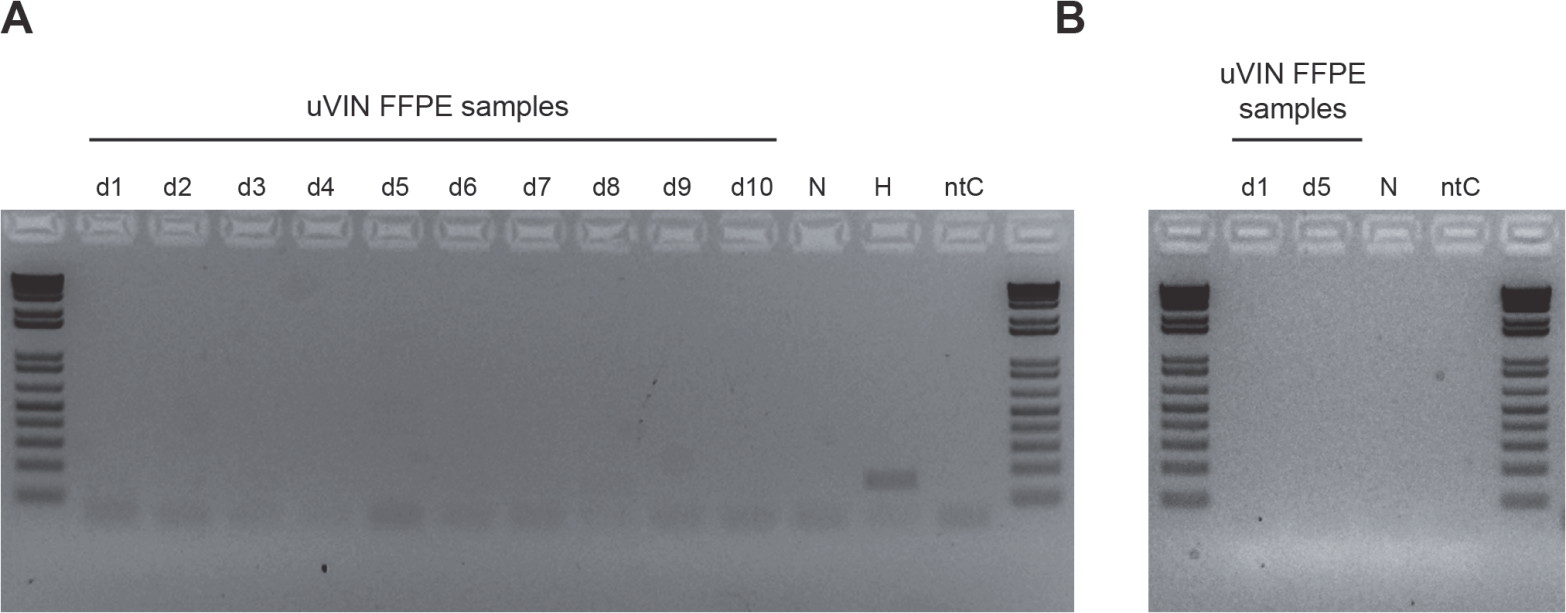
PCR testing for Human herpesvirus 3 (HHV) and Suid herpesvirus 1 (SuHV1). (A) PCR amplification was performed using a primer set designed from a single HHV3 read found in the dVIN sample 1 next-generation sequencing (NGS) data. The expected 163-bp product, identified as HHV3 by confirmatory Sanger sequencing, is only seen in an HHV3-positive cerebrospinal fluid (CSF) sample processed in parallel on the same NGS run, indicating that the single HHV3 read in the dVIN sample 1 NGS dataset was most likely due to cross-contamination. (B) PCR amplification was performed using a primer set designed from two SuHV1 ViroChip probes. Note that the dVIN sample 1 is negative for SuHV1. Abbreviations: L, DNA ladder; d1-d10, dVIN samples 1 through 10; N, normal skin vulvar biopsy; H, HHV3-positive CSF sample;; ntC, no template control.

Next, ten pure (p16^ink4a^)-negative dVIN cases that had no adjacent uVIN or lichen sclerosus (Fig 3A) were selected for viral identification using the ViroChip microarray, which includes probes targeting HPV subtypes 1 through 106. In addition to 10 dVIN case, HPV-positive tissue from a patient with metastatic cervical cancer and vulvar tissue from a healthy individual were used as positive and negative controls, respectively. Microarray Z-score and heat map cluster analysis showed the absence of a papillomavirus signature in the dVIN samples (Table 1 and Fig 3A, cluster on left). In contrast, in the positive control sample, 16 of the top 50 probes by Z-score analysis were derived from HPV-18 (Table 1) and mapped to 4 different regions of the HPV-18 genome (Fig 3B); a corresponding high-intensity HPV-18 cluster was observed by heat map analysis (Fig 3A, magnified cluster on right). Other smaller clusters corresponding to the dVIN samples were found to consist of low-intensity probes from several different papillomavirus types, and follow-up PCR using specific primers targeting each papillomavirus subtype was negative (Fig 3A, two gels on right). PCR using a primer set targeting the highly conserved L1 region of papillomaviruses also confirmed the findings of HPV-18 positivity in the control tissue and HPV negativity in the dVIN cases (Fig 3A, gel on left). Other viruses were not detected by ViroChip in dVIN samples except for a weak, low-intensity signature to a porcine herpesvirus, Suid herpesvirus 1 (SuHV1), in one sample (dVIN sample 1) by Z-score analysis (Table 1). To confirm or refute this potential herpesvirus finding, we used metagenomic next-generation sequencing (NGS) to analyze dVIN sample 1 and a corresponding negative control vulvar tissue sample. A total of 4,173,314 raw NGS reads from dVIN sample 1 and 3,942,852 reads from the negative control were analyzed using the SURPI bioinformatics pipeline for pathogen identification (Table 3). Out the 206 identified viral reads, nearly all of the reads (205 of 206; 99.5%) corresponded to anelloviruses and bacteriophages, considered to non-pathogenic flora (Table 4) [18,27]. A single read corresponding to human herpesvirus 3 (HHV3) was attributed to cross-contamination from an HHV3-positive cerebrospinal fluid sample analyzed in parallel on the same NGS run (Table 4). PCR testing of dVIN sample 1 for HHV3 was negative (Fig 4A). Out of the 3,222,732 identified bacterial reads in dVIN sample 1, the majority of reads (2,942,883, 91%) corresponded to *Pseudomonas* (Table 4). This was attributed to surface bacterial contamination of the non-sterile FFPE blocks, as a high proportion of contaminating *Pseudomonas* reads, as well as reads corresponding to presumed viral flora, were also seen in negative control tissue (Table 4). Further evidence of the absence of SuHV1 in dVIN sample 1 was obtained by subsequent PCR testing using primers designed from ViroChip SuHV1 probes (Fig 4B).

**Table 3:** Number of next-generation sequencing (NGS) reads at each step of the SURPI bioinformatics pipeline for pathogen identification.

Number of Sequence Reads	dVIN-Sample1 (vulvar tissue)	Negative Control (vulvar tissue)	Positive Control (HHV3-positive CSF)
raw reads	4,173,314	3,942,852	9,771,488
reads remaining after preprocessing	4,159,839	3,836,781	8,607,942
non-human reads	4,113,586	1,601,296	930,341
nucleotide alignment to NCBI nt database	3,728,001	1,195,515	168,847
amino acid alignment to viral aa database	19,203	19,204	102,039
# of reads identified as viral	206	330	118,930
# of reads identified as bacterial	3,222,732	1,092,395	9,074

**Table 4:** Viruses and bacteria identified in tissue samples by next-generation sequencing.

Microbes		dVIN-Sample1 (vulvar tissue)	Negative Control (vulvar tissue)	Positive Control (HHV3-positive cerebrospinal fluid)
Virus				
	*Anelloviridae*	30	25	107,433
	*Circoviridae*	0	2	0
	bacteriophage (*Pseudomonas)*	158	72	3
	bacteriophage (other)	17	231	42
	human herpesvirus 3 (HHV3)	1	0	11,452
Bacteria				
	*Pseudomonas*	2,942,883	970,177	7,505
	other bacteria	279,849	122,218	1,569
	% *Pseudomonas*	91%	89%	83%

## Discussion

In this study, we examined the potential association of viral infection with high-grade VIN that gives rise to VSCC. Two precursor lesions are involved in the etiopathogenesis of VSCC. The HPV-related pathway is commonly associated with uVIN as the precursor lesion, and these constitute a small fraction of VSCC cases. The second, more common, etiology of VSCC is related to *p53* mutations, and is associated with dVIN as the precursor lesion. Establishing the diagnosis of dVIN is challenging and interobserver variability is high [28]. Furthermore, although dVIN tissues can be analyzed by p53 immunostaining, interpretation can be problematic and p53 positivity is not seen in all cases [29], making this technique cumbersome for routine clinical practice.

HPV virus is detected by laboratory testing using either PCR or a surrogate marker of HPV infection. Positive p16^ink4a^ immunostaining is correlated with the presence of HPV because the expression of the HPV E7 oncogene leads to inactivation of pRb and a consequent increase of expression of cyclin-dependent kinase inhibitor p16 (p16^INK4a^) [30] Immunostaining for p16^ink4a^, however, can only detect high-risk HPV subtypes. The ViroChip microarray is a broad and highly sensitive technology that is capable not only of detecting all subtypes of HPV, but also other viruses that may be involved in the disease process. In this study, we used the ViroChip microarray and PCR to demonstrate lack of an association between dVIN and viral infection, including infection by any of the HPV subtypes. Although low-intensity clusters of probes representing multiple papillomavirus subtypes were observed sporadically in the microarray data (Fig 3A), follow-up PCR specifically targeting these subtypes was negative, indicating that these spurious clusters most likely arose from probe cross-hybridization [31]. Deep metagenomic next-generation sequencing of one dVIN sample with a weak ViroChip signature for a herpesvirus identified only sequences corresponding to non-pathogenic flora such as *Anelloviridae* and bacteriophages that were detected in both the dVIN and control groups.

While we cannot exclude an infectious etiology for dVIN given the small number of cases analyzed, our study suggests that viral infection is unlikely to play a significant role in the development and pathogenesis of the disease. This has a bearing on the proper diagnosis and treatment of dVIN as compared to uVIN. The diagnostic tools for dVIN currently are limited to histological assessment and p53 immunostaining of biopsies [7]. However, p53 overexpression is not found in all cases of dVIN [29], and given our observed lack of an association with infection by HPV or other viruses, reliable biomarkers for dVIN will still be needed for accurate diagnosis and effective treatment. In addition, antiviral therapies primarily directed at human papillomaviruses such as imiquimod and cidofivir are widely used to treat VIN [32], yet our results suggest that these antivirals would likely not be efficacious in dVIN given lack of a detected viral association. Treatment of dVIN-derived VSCC should instead be focused on surgical excision, local-field irradiation, and adjunct therapies targeting tumor-associated antigens and/or genes.
